# A venda de cigarros avulsos no Brasil entre 2008 e 2019: mais um motivo de preocupação?

**DOI:** 10.1590/0102-311XPT073723

**Published:** 2023-11-10

**Authors:** André Salem Szklo

**Affiliations:** 1 Instituto Nacional de Câncer José Alencar Gomes da Silva, Rio de Janeiro, Brasil.

**Keywords:** Comportamento de Fumar, Política Pública, Tabaco, Inquérito Epidemiológico, Smoking Behavior, Public Policy, Tobacco, Health Survey, Conducta de Fumar, Política Pública, Tabaco, Encuestas Epidemiológicas

## Abstract

No Brasil, a venda de cigarros é permitida apenas em embalagens fechadas com 20 unidades. Avaliou-se a evolução ao longo do tempo da proporção de fumantes adultos que adquiriram cigarros industrializados avulsos na última compra. Utilizaram-se os dados da *Pesquisa Especial de Tabagismo* conduzida em 2008 e da *Pesquisa Nacional de Saúde* conduzida em 2013 e 2019. Modelo linear generalizado foi usado para calcular as diferenças na proporção de compra de cigarros avulsos entre os anos das pesquisas, ajustadas por variáveis sociodemográficas e de comportamento de fumar. Considerando 2013 como ano de referência, as diferenças relativas entre as proporções foram, respectivamente, -15,3% (valor de p ajustado ≤ 0,05) na comparação com 2008, e +13,3 (valor de p ajustado = 0,08) na comparação com 2019. Cerca de 20% dos jovens adultos fumantes relataram comprar cigarro avulso em 2019 e a diferença na proporção de compra de cigarro avulso entre indivíduos de 18 a 24 anos e aqueles mais velhos provavelmente aumentou entre 2013 e 2019 (valor de p interação ajustado = 0,08). Há motivos de preocupação, pois o fortalecimento da política tributária entre 2008 e 2013 foi acompanhado de um aumento na proporção de compra de cigarros avulsos. Apesar da queda do preço real do maço de cigarros a partir de 2017, um contexto de baixa efetividade de implementação de outras medidas antitabagismo acentuou provavelmente a diferença da proporção de compra de cigarros avulsos entre jovens e adultos. A presença permanente do cigarro avulso como modalidade de aquisição contribui para que subgrupos populacionais mais vulneráveis do ponto de vista econômico se tornem e/ou permaneçam dependentes do comportamento de fumar.

## Abstract (en)

In Brazil, the sale of cigarettes is only allowed in closed packages with 20 units. The evolution over time of the proportion of adult smokers who purchased single manufactured cigarettes in their last purchase was evaluated. Data from the *Global Adult Tobacco Survey* conducted in 2008 and the *Brazilian National Health Survey* conducted in 2013 and 2019 were used. A generalized linear model was used to calculate the differences in the proportion of single cigarette purchases between the survey years, adjusted for sociodemographic and smoking behavior variables. Considering 2013 as the reference year, the relative differences in the proportions were, respectively, -15.3% (adjusted p-value ≤ 0.05) when compared to 2008, and +13.3 (adjusted p-value = 0.08) when compared to 2019. Approximately 20% of young adult smokers reported buying single cigarettes in 2019 and the difference in the proportion of single cigarette purchases between individuals aged 18 to 24 and those older likely increased from 2013 to 2019 (adjusted interaction p-value = 0.08). There are reasons for concern, as the strengthening of tax policy from 2008 to 2013 was accompanied by an increase in the proportion of purchases of single cigarettes. Despite the decrease in the real price of cigarette packs from 2017, a context of low effectiveness of implementation of other anti-smoking measures likely accentuated the difference in the proportion of purchase of single cigarettes between young people and adults. The continued presence of single cigarettes as a mode of acquisition contributes to economically vulnerable population subgroups becoming and/or remaining dependent on smoking behavior.

## Resumen

En Brasil, la venta de cigarrillos está permitida solo en paquetes cerrados que llevan veinte unidades cada. Se evaluó la evolución a lo largo del tiempo de la proporción de fumadores adultos que adquirieron cigarrillos industrializados sueltos en la última compra. Se utilizaron los datos de la *Encuesta Especial de Tabaquismo* realizada en 2008 y de la *Encuesta Nacional de Salud* realizada en 2013 y 2019. Se utilizó un modelo lineal generalizado para calcular las diferencias en la proporción de compra de cigarrillos sueltos entre los años de las encuestas, ajustada por variables sociodemográficas y de comportamiento de fumar. Considerando 2013 como año de referencia, las diferencias relativas entre las proporciones fueron, respectivamente, -15,3% (valor de p ajustado ≤ 0,05) en comparación con 2008, y +13,3 (valor de p ajustado = 0,08) en comparación con 2019. Cerca del 20% de los fumadores jóvenes adultos informaron haber comprado cigarrillos sueltos en 2019, y es probable que la diferencia en la proporción de la compra de cigarrillos sueltos entre indivíduos de 18 y 24 años y aquellos más adultos haya aumentado entre 2013 y 2019 (valor de p de interacción ajustado = 0,08). Hay motivos de preocupación, ya que el fortalecimiento de la política fiscal entre 2008 y 2013 estuvo acompañado de un aumento en la proporción de compra de cigarrillos sueltos. Aunque el precio real del paquete de cigarrillos a partir de 2017, un contexto de baja efectividad en la implementación de otras medidas contra el tabaquismo probablemente acentuó la diferencia en la proporción de compra de cigarrillos sueltos entre jóvenes y adultos. La presencia continua de cigarrillos sueltos como modalidad de adquisición contribuye a que los subgrupos de la población económicamente más vulnerables se vuelvan y/o permanezcan dependientes del comportamiento de fumar.

## Introdução

A política de controle do tabaco do Brasil é norteada pelos objetivos, princípios e obrigações presentes na Convenção-Quadro para o Controle do Tabaco (CQCT), primeiro tratado internacional de saúde pública negociado sob a coordenação da Organização Mundial da Saúde (OMS) e ratificado pelo país em 2005 ^1^.

Entre uma série de medidas relativas à redução da demanda de tabaco, aquela relacionada ao aumento de preços e impostos é considerada a mais eficaz para que diversos segmentos da população, em particular os jovens que não têm tantos recursos financeiros, reduzam/cessem o consumo de tabaco (Artigo 6/CQCT) ^1^^,^^2^. Segundo a normativa da Receita Federal do Brasil, que dispõe sobre a incidência do imposto sobre produtos industrializados (IPI), a venda de cigarros é permitida apenas em embalagens fechadas contendo 20 unidades ^3^. A venda ilegal de cigarros avulsos acaba sendo, portanto, uma opção econômica mais vantajosa para os subgrupos populacionais com menor poder aquisitivo e que não fumam muitos cigarros por dia, o que favorece a iniciação/manutenção ao/do tabagismo ^2^^,^^4^^,^^5^^,^^6^.

Já a utilização das embalagens dos produtos derivados do tabaco é também uma importante medida para comunicar à população os reais efeitos negativos do tabagismo (Artigo 11/CQCT) ^1^^,^^7^^,^^8^. Nesse sentido, a Agência Nacional de Vigilância Sanitária (Anvisa) apenas concede o registro da marca do fabricante a partir de um exemplar da embalagem do produto derivado do tabaco destinado à comercialização, além de regulamentar como as advertências sanitárias devem ser impressas nos pacotes ^9^.

Tal como preconizado no Artigo 20/CQCT ^1^, o Brasil conta com um sistema de monitoramento da epidemia do tabagismo composto por uma série de perguntas sobre o comportamento de fumar inseridas em pesquisas periódicas nacionais realizadas tanto na população jovem quanto na adulta ^10^^,^^11^^,^^12^. Diferentemente da queda na proporção de fumantes entre jovens adultos observada entre 2008 e 2013 (13,6% em 2008 *versus* 10,5% em 2013) ^10^^,^^11^^,^^13^, dados mais recentes apontam para uma estabilidade (10,6% em 2019) ^12^. Esse fato sinaliza um provável enfraquecimento na efetividade da implementação das diversas medidas legislativas e educacionais antitabagismo voltadas para estimular a cessação e reduzir a iniciação ao fumo ^14^^,^^15^^,^^16^^,^^17^^,^^18^^,^^19^, tal como, por exemplo, a falta de reajuste, desde 2017, no preço mínimo do cigarro estabelecido por lei e nas alíquotas do imposto que incidem sobre os produtos derivados do tabaco ^3^^,^^14^^,^^17^.

O objetivo deste estudo foi, portanto, avaliar a evolução ao longo do tempo da proporção de compra de cigarro avulso pelo fumante, estratificada por variáveis sociodemográficas e de comportamento de fumar. Até onde os autores sabem, não existe nenhum estudo que tenha analisado, a partir de inquéritos nacionais seriados, o tema do cumprimento/descumprimento do conjunto de legislações que proíbem a venda de cigarros avulsos, i.e., que proíbem a venda de cigarros fora das embalagens ^3^^,^^9^.

## Metodologia

Este artigo utiliza os dados sociodemográficos e de comportamento de fumar da *Pesquisa Especial de Tabagismo* (PETab) conduzida em 2008 como um suplemento da *Pesquisa Nacional por Amostra de Domicílio*s (PNAD) ^10^, além dos dados da *Pesquisa Nacional de Saúde* (PNS), conduzida em 2013 e 2019 ^11^^,^^13^. Todos esses estudos foram realizados pelo Instituto Brasileiro de Geografia e Estatística (IBGE), com uso de planos amostrais cujo objetivo era o representar a população brasileira de 15 anos ou mais (PETab e PNS 2019) ou de 18 anos ou mais (PNS 2013). Mais detalhes sobre as metodologias dessas pesquisas podem ser encontrados em publicações recentes ^10^^,^^11^^,^^13^.

A pergunta que definiu a compra de cigarro avulso pelo fumante tanto na PETab quanto nas edições da PNS foi a seguinte: “Na última vez em que o(a) Sr(a) comprou cigarros para uso próprio, quantos cigarros comprou?”, sendo que as opções de resposta eram “cigarros”, “maços”, “pacotes” e “nunca comprei cigarros para uso próprio”. Essa pergunta foi feita apenas para aqueles indivíduos que afirmaram fumar, atualmente, cigarros industrializados. Após excluir os fumantes que afirmaram nunca ter comprado cigarros para uso próprio, aqueles que compraram por “maço” ou “pacote” foram agrupados de forma a criar a variável dicotômica “compra de cigarro avulso” (“não” *versus* “sim”). Vale a pena assinalar que os indivíduos que compraram cigarros para uso próprio também forneceram a informação em relação à quantidade adquirida em termos, respectivamente, de cigarros avulsos (ou “maços + cigarros por maço”, ou “pacotes + maços por pacotes”). Sendo assim, indivíduos que afirmaram ter comprado “cigarros” na última compra em número igual ou superior a múltiplos de 20 foram reclassificados como compradores de “maço ou pacote” ^3^, partindo do pressuposto de que não seria razoável pagar mais caro por 20 cigarros avulsos equivalentes a um maço contendo 20 cigarros ^3^^,^^20^.

As análises do artigo foram restritas, portanto, aos fumantes de cigarros industrializados com 18 anos de idade ou mais que em algum momento da vida já compraram seus próprios cigarros. Informações sociodemográficas e de comportamento de fumar amplamente descritas na literatura como relacionadas ao uso de cigarros avulsos ^4^^,^^6^^,^^21^^,^^22^ foram utilizadas nas análises como variáveis dicotômicas, tal como segue: (1) sexo, classificado como mulher ou homem; (2) idade, dividida entre 18 e 24 anos *versus* 25 anos ou mais; (3) escolaridade, classificada como Ensino Fundamental completo ou mais *versus* Ensino Fundamental incompleto; (4) região de residência, agrupada entre Norte (compreende os estados de Rondônia, Acre, Amazonas, Roraima, Pará, Amapá e Tocantins) e Nordeste (compreende os estados do Maranhão, Piauí, Ceará, Rio Grande do Norte, Paraíba, Pernambuco, Alagoas, Sergipe e Bahia) *versus* Centro-oeste (compreende os estados de Mato Grosso, Mato Grosso do Sul e Goiás e o Distrito Federal), Sudeste (compreende os estados de Minas Gerais, Espírito Santo, Rio de Janeiro e São Paulo) e Sul (compreende os estados do Paraná, Santa Catarina e Rio Grande do Sul), a partir também de análise preliminar dos bancos de dados; (5) frequência de consumo de cigarros, definida a partir da pergunta “Atualmente, o(a) Sr(a) fuma algum produto do tabaco?” e classificada com base nas respostas “sim, diariamente” ou “sim, ocasionalmente”. Além disso, indivíduos que fumam cigarros industrializados ilegais (*versus* legais) foram separados a partir de metodologia baseada no critério do nome autorreportado da marca de cigarros da última compra (informação disponível apenas para o ano de 2019) ^9^^,^^23^.

Inicialmente, calculou-se, para os anos das pesquisas (2008, 2013 e 2019), a distribuição dos fumantes de cigarros industrializados estratificada pelas variáveis sociodemográficas e de comportamento de fumar. Para essa análise estatística descritiva, o teste qui-quadrado foi utilizado para comparar as correspondentes proporções entre os anos das pesquisas (2008 *versus* 2013 e 2013 *versus* 2019).

Posteriormente, como a variável desfecho “compra de cigarro avulso” era dicotômica, foi utilizado modelo linear generalizado com distribuição binomial e função de ligação identidade para avaliar a diferença absoluta (obtida diretamente do coeficiente de regressão do modelo) da compra de cigarro avulso ^24^. Usou-se a função de ligação logarítmica para avaliar também a diferença relativa (obtida a partir da exponencial do coeficiente de regressão do modelo - 1) ^24^. Foram calculadas as respectivas diferenças brutas e ajustadas para o ano da pesquisa, variáveis sociodemográficas e de comportamento de fumar. Em razão das diversas etapas de implementação das medidas legislativas e educacionais antitabagismo ao longo do tempo, voltadas para estimular a cessação e reduzir a iniciação ao fumo ^1^^,^^3^^,^^9^^,^^14^^,^^15^^,^^16^^,^^17^^,^^18^^,^^19^, as diferenças de proporção de compra de cigarro avulso entre as categorias das variáveis sociodemográficas e de comportamento de fumar poderiam não ser homogêneas segundo os anos das pesquisas. Sendo assim, foram adicionados, inicialmente, aos modelos das diferenças absoluta e relativa contendo as informações agrupadas das três pesquisas ^10^^,^^11^^,^^13^ os termos de interação “sexo*ano da pesquisa” (ou “idade*ano da pesquisa”, “escolaridade*ano da pesquisa”, “região*ano da pesquisa”, “consumo diário*ano da pesquisa”), fixando 2013 como ano de referência para a variável ano da pesquisa. No entanto, eles não foram mantidos nos respectivos modelos finais ajustados por não terem sido estatisticamente significativos, i.e., com valor de p ≤ 0,05.

Ademais, foram estimados, para cada ano de pesquisa, os números totais de fumantes de cigarros avulsos e o número total de cigarros avulsos comprados, estratificados pelas variáveis sociodemográficas e de comportamento de fumar. Para estimar o número total de cigarros avulsos comprados por ano, foi necessário ponderar pela informação relativa ao consumo médio diário de cigarros dos fumantes, cuja última compra correspondeu a uma aquisição de cigarro avulso, multiplicada por 365,25. Para tal, utilizou-se a resposta à pergunta “Em média, quantos cigarros industrializados o(a) Sr(a) fuma por dia ou por semana atualmente?”. O consumo médio diário de cigarros para aqueles que responderam “por semana” foi calculado dividindo a informação fornecida por 7.

Finalmente, apenas para o ano de 2019, foram estimadas também a proporção de consumo de cigarro ilegal entre os fumantes de cigarro industrializado, a proporção de compra de cigarro avulso estratificada por *status* de compra de cigarro ilegal (assim como as respectivas diferenças brutas absoluta e relativa) e os números totais de fumantes e de cigarros avulsos comprados.

Todas as análises foram realizadas com o programa estatístico Stata, versão 15.0 (https://www.stata.com), considerando o desenho amostral complexo das pesquisas.

## Resultados

A Tabela 1 mostra que a população de fumantes de cigarro industrializado (16,3 milhões em 2008, 17,5 milhões em 2013 e 15,5 milhões em 2019) está concentrada entre os homens, apesar de ter havido uma redução estatisticamente significativa, com valor de p ≤ 0,05, entre 2013 e 2019. Nota-se, ainda, uma redução gradual na participação dos jovens adultos (18-24 anos), principalmente de 2008 a 2013, na população de fumantes de cigarros industrializados. No que diz respeito à escolaridade, enquanto em 2008 a população de fumantes estava mais concentrada entre aqueles de menor escolaridade, a partir de 2013 houve uma inversão nessa realidade, a qual se acentuou em 2019. Da mesma forma, observou-se para o período mais recente menor participação da população residente nas regiões Norte e Nordeste entre os fumantes de cigarro industrializado. Quando analisamos a proporção de indivíduos que relataram fumar cigarros diariamente, os quais representam a ampla maioria da população de fumantes, percebemos uma queda da sua contribuição relativa entre 2008 e 2013, seguida de um aumento entre 2013 e 2019. Finalmente, em 2019, cerca de 1/3 dos fumantes de cigarro industrializado relataram ter adquirido na última compra uma marca de cigarros de origem ilegal.

**Tabela 1:** Distribuição de fumantes de cigarros industrializados ^#,##^, segundo variáveis sociodemográficas e de comportamento de fumar. Indivíduos com idade ≥ 18 anos. Brasil, 2008, 2013 e 2019.

Características selecionadas	Fumantes adultos (%) ^#,##^		
	PETab 2008 (N = 16,3*106) ^#,##^	PNS 2013 (N = 17,5*106) ^#,##^	PNS 2019 (N = 15,5*106) ^#,##^
Sexo			
Mulher	40,8	39,0	42,9
Homem	59,2	61,0 ^###^	57,2 ^###^
Faixa etária (anos)			
25 ou mais	85,1	87,4	88,1
18-24	14,9 ^###^	12,6 ^###^	10,9
Escolaridade			
Ensino Fundamental completo ou mais	48,0	53,1	57,6
Ensino Fundamental incompleto	52,0 ^###^	46,8 ^###^	42,4 ^###^
Região de residência			
Sudeste/Sul/Centro-oeste	71,9	71,7	75,3
Norte/Nordeste	28,1	28,3 ^###^	24,7 ^###^
Fumante diário			
Sim	89,7	87,7	92,1
Não	10,3 ^###^	12,3 ^###^	7,9 ^###^
Consumo de cigarros ilegais ^§^			
Não	n.d.	n.d.	64,9
Sim	n.d.	n.d.	35,1

n.d.: não disponível; PETab: *Pesquisa Especial de Tabagismo*; PNS: *Pesquisa Nacional de Saúde*.

^#^ Prevalência de fumantes (cigarros industrializados): 2008 (15,3%), 2013 (12,5%), 2019 (9,9%);

^##^ Indivíduos que que afirmaram nunca ter comprado cigarros para uso próprio foram excluídos da análise (2008, 9,8%; 2013, 3,1%; 2019, 1,7%);

^###^ Valor de p ≤ 0,05 na comparação das proporções de indivíduos em cada categoria da variável selecionada entre 2008 e 2013 (ou entre 2013 e 2019);

^§^ Indivíduos que fumam cigarros industrializados ilegais (*versus* legais) foram separados a partir de metodologia baseada no critério do nome autorreportado da marca de cigarros da última compra (disponível apenas para o ano de 2019).

Os dados brutos apresentados na Tabela 2 mostram que a proporção de indivíduos que adquiriram cigarros avulsos na última compra foi menor em 2008 quando comparada à de 2013, tanto na diferença absoluta quanto na relativa (valores de p ≤ 0,05). No entanto, não foram observadas diferenças estatisticamente significativas na comparação para o período mais recente. Fumantes do sexo masculino, indivíduos com menos de 25 anos de idade, com Ensino Fundamental incompleto, residentes nas regiões Norte ou Nordeste e fumantes ocasionais apresentaram proporções de compra de cigarro avulso superiores às suas respectivas categorias de comparação (diferenças absolutas e relativas brutas, respectivamente, estatisticamente significativas com valores de p ≤ 0,05). Todas as diferenças entre as categorias das variáveis sociodemográficas e de comportamento de fumar foram homogêneas segundo os anos de pesquisa (valores de p brutos dos respectivos termos de interação ≥ 0,10; Tabela 3). A maior diferença bruta na escala absoluta foi observada na comparação entre fumantes ocasionais e fumantes diários (+26,15%); e a maior diferença bruta na escala relativa foi identificada na comparação entre residentes das regiões Norte e Nordeste *versus* moradores das outras regiões brasileiras (+384,51%). Vale a pena assinalar que, em 2019, não foram encontradas diferenças absolutas ou relativas na proporção de compra de cigarros avulsos segundo status de compra de cigarro ilegal.

**Tabela 2:** Diferenças brutas e ajustadas na proporção de indivíduos que relataram ter adquirido cigarros avulsos na última compra, segundo ano da pesquisa, variáveis sociodemográficas e de comportamento de fumar. Indivíduos com idade ≥ 18 anos. Brasil, 2008, 2013 e 2019.

Características selecionadas	Indivíduos que relataram ter adquirido cigarros avulsos na última compra				
	Cigarros avulsos	Diferença bruta ^#^		Diferença ajustada ^##^	
	% (IC95%)	Absoluta [% (IC95%)]	Relativa [% (IC95%)]	Absoluta [% (IC95%)]	Relativa [% (IC95%)]
Ano da pesquisa					
2008	8,9 (7,8; 10,1)	-1,77 (-3,30; -0,24) ^###^	-16,66 (-28,69; -2,56) ^###^	-0,41 (-1,45; 0,63)	-15,31 (-26,91; -1,87) ^###^
2013	10,6 (9,5; 11,9)	Referência	Referência	Referência	Referência
2019	10,3 (9,3; 11,4)	-0,34 (-1,87; 1,19)	-3,19 (-16,32; 12,00)	1,01 (-0,12; 2,13)	13,31 (-1,29; 30,07)
Sexo					
Mulher	8,7 (7,8; 9,6)	Referência	Referência	Referência	Referência
Homem	10,8 (10,0; 11,8)	2,20 (0,99; 3,41) ^###^	25,42 (10,45; 42,43) ^###^	0,38 (-0,48; 1,23)	1,57 (-10,07; 14,71)
Faixa etária (anos)					
25 ou mais	8,8 (8,2; 9,4)	Referência	Referência	Referência	Referência
18-24	17,8 (15,4; 20,5)	9,03 (6,47; 11,60) ^###^	102,79 (74,00; 136,88) ^###^	4,68 (2,37; 6,99) ^###^	61,45 (38,84; 87,74) ^###^
Escolaridade					
Ensino Fundamental completo ou mais	8,9 (8,1; 9,7)	Referência	Referência	Referência	Referência
Ensino Fundamental incompleto	11,2 (10,3; 12,2)	2,32 (1,09; 3,55) ^###^	26,17 (11,55; 42,72) ^###^	0,48 (-0,39; 1,34)	12,45 (1,01; 26,44) ^###^
Região de residência					
Sudeste/Sul/Centro-oeste	4,9 (4,3; 5,5)	Referência	Referência	Referência	Referência
Norte/Nordeste	23,6 (22,1; 25,2)	18,74 (17,12; 20,36) ^###^	384,51 (324,32; 453,25) ^###^	15,33 (13,65; 17,02) ^###^	290,71 (239,27; 349,94) ^###^
Fumante diário					
Sim	7,3 (6,7; 7,9)	Referência	Referência	Referência	Referência
Não	33,4 (30,4; 36,5)	26,15 (23,11; 29,19) ^###^	360,02 (308,68; 417,80) ^###^	21,27 (18,07; 24,48) ^###^	217,09 (179,0; 260,0) ^###^
Consumo de cigarros ilegais ^§^					
Não	10,1 (9,0; 11,4)	Referência	Referência	-	-
Sim	10,3 (8,6; 12,2)	0,13 (-1,95; 2,22)	1,31 (-17,42; 24,86)	-	-

IC95%: intervalo de 95% de confiança.

Nota: indivíduos que afirmaram nunca ter comprado cigarros para uso próprio foram excluídos da análise (2008, 9,8%; 2013, 3,1%; 2019, 1,7%).

^#^ Modelo linear generalizado tendo como família a distribuição binomial e função de ligação identidade (diferença absoluta) ou logarítmica (diferença relativa);

^##^ Modelo linear generalizado tendo como família a distribuição binomial e função de ligação identidade (diferença absoluta) ou logarítmica (diferença relativa), ajustado simultaneamente por sexo, idade, escolaridade, região de residência, frequência de consumo;

^###^ Valor de p ≤ 0,05;

^§^ Indivíduos que fumam cigarros industrializados ilegais (*versus* legais) foram separados a partir de metodologia baseada no critério do nome autorreportado da marca de cigarros da última compra (disponível apenas para o ano de 2019).

**Tabela 3:** Valores de p ^#,##^ dos termos de interação “sexo*ano da pesquisa” (ou “idade*ano da pesquisa”, “escolaridade*ano da pesquisa”, “região*ano da pesquisa”, “consumo diário*ano da pesquisa”) das diferenças brutas e ajustadas na proporção de indivíduos que relataram ter adquirido cigarro avulso na última compra, segundo variáveis sociodemográficas e de comportamento de fumar. Indivíduos com idade ≥ 18 anos. Brasil, 2008, 2013 e 2019.

Características selecionadas	Ano da pesquisa							Todos os anos			
	PETab 2008			PNS 2013	PNS 2019						
	Cigarro avulso	Valor de p termo de interação (2008-2013) ^#^		Cigarro avulso	Cigarro avulso	Valor de p termo de interação (2013-2019) ^#^		Valor de p termo de interação (2008-2013) ^##^		Valor de p termo de interação (2013-2019) ^##^	
	% (IC95%)	Absoluta bruta	Relativa bruta	% (IC95%)	% (IC95%)	Absoluta bruta	Relativa bruta	Absoluta ajustada	Relativa ajustada	Absoluta ajustada	Relativa ajustada
Sexo											
Mulher	8,9 (7,4; 10,6)	0,234	0,309	9,1 (7,4; 11,0)	8,5 (7,2; 10,0)	0,672	0,645	0,602	0,445	0,412	0,362
Homem	9,9 (8,4; 11,5)			11,7 (10,1; 13,4)	11,7 (10,4; 13,1)						
Faixa etária (anos)											
25 ou mais	8,1 (7,1; 9,3)	0,523	0,230	9,8 (8,6; 11,0)	9,0 (8,0; 10,0)	0,095	0,102	0,531	0,628	0,075	0,082
18-24	17,0 (13,5; 21,0)			16,8 (12,3; 22,2)	21,2 (17,2; 26,0)						
Escolaridade											
Ensino Fundamental completo ou mais	8,6 (7,2; 10,4)	0,452	0,608	9,4 (8,0; 11,1)	8,9 (7,8; 10,3)	0,711	0,683	0,407	0,489	0,886	0,965
Ensino Fundamental incompleto	10,2 (8,8; 11,9)			12,0 (10,3; 14,0)	12,2 (10,6; 13,9)						
Região de residência											
Sudeste/Sul/Centro-oeste	4,7 (3,8; 5,7)	0,278	0,604	5,3 (4,3; 6,5)	5,2 (4,3; 6,2)	0,374	0,580	0,153	0,665	0,368	0,584
Norte/Nordeste	21,4 (18,8; 24,4)			24,3 (21,6; 27,2)	26,0 (23,6; 28,5)						
Fumante diário											
Sim	7,1 (6,1; 8,2)	0,124	0,599	7,1 (6,1; 8,3)	8,2 (7,3; 9,2)	0,614	0,255	0,094	0,424	0,528	0,172
Não	29,7 (24,9; 35,0)			35,7 (30,8; 40,9)	34,8 (29,4; 40,6)						

IC95%: intervalo de 95% de confiança; PETab: *Pesquisa Especial de Tabagismo*; PNS: *Pesquisa Nacional de Saúde*.

Nota: indivíduos que afirmaram nunca ter comprado cigarros para uso próprio foram excluídos da análise (2008, 9,8%; 2013, 3,1%; 2019, 1,7%).

^#^Modelo linear generalizado tendo como família a distribuição binomial e função de ligação identidade (diferença absoluta) ou logarítmica (diferença relativa);

^##^Modelo linear generalizado tendo como família a distribuição binomial e função de ligação identidade (diferença absoluta) ou logarítmica (diferença relativa), ajustado simultaneamente por sexo, idade, escolaridade, região de residência, frequência de consumo e termos de interação aditiva (ou multiplicativa) “sexo*ano da pesquisa” (ou “idade*ano da pesquisa”, “escolaridade*ano da pesquisa”, “região*ano da pesquisa”, “consumo diário*ano da pesquisa”), fixando 2013 como ano de referência para a variável ano da pesquisa.

Ainda, a Tabela 2 mostra que as diferenças absolutas e relativas ajustadas apontam, embora com magnitudes inferiores, na mesma direção das diferenças brutas para jovens adultos (+4,68% e +61,45%, respectivamente), indivíduos residentes nas regiões Norte e Nordeste (+15,33% e +290,71%, respectivamente) e fumantes ocasionais (+21,27% e +217,09%, respectivamente) (*versus* suas respectivas categorias de comparação). As diferenças absoluta e relativa ajustadas entre homens e mulheres não foram estatisticamente significativas. As diferenças ajustadas entre os anos da pesquisa 2013 e 2008, e entre os indivíduos de “alta” e “baixa” escolaridade, permaneceram estatisticamente significativas apenas na escala relativa (-15,31% e +12,45%, respectivamente). É importante mencionar que, embora as diferenças absoluta e relativa entre os anos de pesquisa 2013 e 2019 sugiram um aumento na proporção de compra de cigarros avulsos, elas não foram estatisticamente significativas (valores de p ajustados = 0,08, não mostrados em tabela).

Todas as diferenças entre as categorias das variáveis sociodemográficas e de comportamento de fumar foram homogêneas segundo os anos de pesquisa (valores de p ajustados dos respectivos termos de interação > 0,05; Tabela 3), embora os resultados indiquem que a diferença absoluta na proporção de compra de cigarros avulsos entre os fumantes ocasionais e fumantes diários foi menor em 2008 (*versus* 2013) (valor de p ajustado do termo de interação = 0,094). Além disso, as diferenças absolutas e relativas na proporção de compra de cigarros avulsos entre os jovens adultos e indivíduos com mais de 24 anos sugerem um aumento entre 2013 e 2019 (valores de p ajustados dos respectivos termos de interação = 0,075 e 0,082, respectivamente).

A Tabela 4 mostra que a estimativa pontual do número absoluto de fumantes que adquiriram cigarros industrializados em formato avulso na última compra aumentou entre 2008 e 2013, reduzindo posteriormente entre 2013 e 2019, quando atingiu 1,6 milhão de fumantes. No que diz respeito ao consumo total anual estimado de cigarros avulsos, foi observada uma queda entre 2008 e 2019, chegando a cerca de 2,7 bilhões de unidades adquiridas em 2019. É interessante notar que, apesar de ter sido observada uma redução na participação dos jovens adultos na população de fumantes de cigarros industrializados (Tabela 1), o total desses jovens que adquiriram cigarros avulsos permaneceu praticamente estável - em cerca de 360 mil - entre 2013 e 2019. Já o consumo anual total de cigarros caiu de 630 milhões para 480 milhões de unidades, respectivamente, entre 2013 e 2019. Finalmente, a Tabela 4 mostra que, em 2019, 550 mil indivíduos estavam em situação de “dupla ilegalidade” ao adquirir cigarros avulsos de marcas ilegais, representando cerca de 1,1 bilhão de unidades por ano.

**Tabela 4:** Total de indivíduos que relataram ter adquirido cigarro avulso na última compra e número total de cigarros avulsos consumidos por ano, segundo variáveis sociodemográficas e de comportamento de fumar. Brasil, 2008, 2013 e 2019.

Características selecionadas	Indivíduos que relataram ter adquirido cigarro avulso na última compra					
	PETab 2008		PNS 2013		PNS 2019	
	Número de indivíduos (N*10^6^)	Número de cigarros (N*10^9^) ^#^	Número de indivíduos (N*10^6^)	Número de cigarros (N*10^9^) ^#^	Número de indivíduos (N*10^6^)	Número de cigarros (N*10^9^) ^#^
Total	1,44	3,43	1,86	2,86	1,60	2,74
Sexo						
Mulher	0,56	1,11	0,62	1,00	0,56	0,93
Homem	0,88	2,32	1,24	1,86	1,04	1,81
Faixa etária (anos)						
25 ou mais	1,04	2,77	1,50	2,23	1,24	2,26
18-24	0,40	0,66	0,37	0,63	0,36	0,48
Escolaridade						
Ensino Fundamental completo ou mais	0,63	1,65	0,88	1,30	0,80	1,33
Ensino Fundamental incompleto	0,81	1,78	0,99	1,56	0,80	1,41
Região de residência						
Sudeste/Sul/Centro-oeste	0,48	1,52	0,66	1,11	0,60	1,25
Norte/Nordeste	0,96	1,91	1,20	1,75	0,99	1,50
Fumante diário						
Sim	0,95	3,24	1,09	2,40	1,17	2,66
Não	0,49	0,19	0,77	0,46	0,43	0,08
Consumo cigarros ilegais ^##^						
Não	n.d.	n.d.	n.d.	n.d.	1,01	1,63
Sim	n.d.	n.d.	n.d.	n.d.	0,55	1,06

n.d.: não disponível; PETab: *Pesquisa Especial de Tabagismo*; PNS: *Pesquisa Nacional de Saúde*.

^#^ Todas as estimativas foram ponderadas pelo consumo médio diário de cigarros multiplicado por 365,25;

^##^ Indivíduos que fumam cigarros industrializados ilegais (*versus* legais) foram separados a partir de metodologia baseada no critério do nome autorreportado da marca de cigarros da última compra (disponível apenas para o ano de 2019).

## Discussão

O dado bruto da proporção de cigarros avulsos adquiridos no Brasil mostrou um aumento entre 2008 e 2013, seguido posteriormente de uma estabilidade até 2019. A maior parte da população de fumantes de cigarros industrializados brasileira está concentrada nos subgrupos populacionais que recorrem menos ao uso do cigarro avulso mais barato, seja porque suas condições socioeconômicas são relativamente melhores ou porque têm uma frequência de consumo mais elevada (e.g., fumantes com 25 anos ou mais, com pelo menos Ensino Fundamental completo, residentes em regiões com maior concentração de renda e fumantes diários). Mesmo assim, os resultados encontrados sugerem que a reforma tributária que entrou em vigor em 2012 ^3^ e elevou o preço final do cigarro ao consumidor em 146% entre 2008 e 2014 ^25^ pode ter contribuído para que a proporção total de compra de cigarro avulso em 2013 chegasse a quase 11% no país. Desde 2017, contudo, o Brasil observa um congelamento da sua política tributária referente às alíquotas que incidem sobre os produtos derivados do tabaco e ao preço mínimo estabelecido por lei, acarretando queda do preço real do maço do cigarro e, consequentemente, aumento da sua acessibilidade ^3^^,^^14^^,^^17^. Nesse sentido, chama a atenção que o dado bruto da proporção de compra de cigarros avulsos tenha permanecido estável entre 2013 e 2019, sobretudo considerando a mudança na distribuição das variáveis sociodemográficas e de comportamento de fumar da população de fumantes de cigarros industrializados nesse período, com uma queda na participação relativa justamente dos subgrupos populacionais que consomem maior proporção de cigarros avulsos. Por outro lado, as análises ajustadas indicam um aumento pontual para o período mais recente na proporção de compra de cigarros avulsos, no limite da significância estatística, tanto na escala absoluta (+1,0%) quanto na relativa (+13,3%). Os dados ajustados sugerem, ainda, um aumento na compra de cigarros avulsos entre os jovens adultos, quando comparados aos mais velhos, entre 2013 e 2019.

O crescimento do consumo de cigarros de origem ilegal observado no Brasil após a reforma tributária de 2011 ^18^^,^^25^, chegando a uma fatia de 40% do mercado brasileiro em 2019 ^17^, que tem a contribuição importante de determinantes macrossociais, tais como a presença da corrupção, impunidade e/ou aceitação da ilegalidade pela sociedade ^26^, pode de certa forma ter criado um terreno fértil para que o descumprimento em geral das leis antitabaco se acentuasse no país. Um estudo recente que analisou a baixa efetividade da implementação do Artigo 16/CQCT, relacionado à venda de cigarros para menores de idade no país, identificou aumento da proporção de aquisição de cigarros para uso próprio entre adolescentes, de forma regular e ilegal, em estabelecimentos comerciais autorizados, entre 2015 e 2019 (81,1% *versus* 89,6%) ^19^. Também foi observada nesse mesmo estudo “a estreita convivência de uma dupla ilegalidade”, em que 70% dos jovens entre 13 e 17 anos relataram que a modalidade mais utilizada de comprar seus próprios cigarros em estabelecimentos comerciais autorizados, tais como bar, banca de jornal e supermercado, foi a compra de cigarro avulso ^19^. Vale a pena assinalar que os resultados apresentados para a PNS de 2019 também apontaram para a convivência de duas ilegalidades, haja vista que aproximadamente 10% dos indivíduos que relataram consumir marcas de cigarros que não pagam impostos no país compraram cigarros avulsos e na mesma proporção dos indivíduos que relataram consumir cigarros fabricados legalmente no país. Estudos futuros, tanto quantitativos quanto qualitativos, são necessários, portanto, para entender melhor como está ocorrendo essa dinâmica da retroalimentação da ilegalidade no país como um todo e em âmbito regional. Tais achados reforçam, ainda, a necessidade de fortalecer a implementação efetiva do Artigo 15/CQCT ^1^, bem como do Protocolo para Eliminar o Comércio Ilícito de Produtos de Tabaco, ao qual o Brasil é também legalmente vinculado ^27^, ambos voltados à redução da oferta de cigarros de origem ilegal.

O caminho para tentar adquirir cigarro avulso no país (e, ao fazê-lo, ser bem-sucedido) reflete, ainda, uma série de eventos oriundos da baixa efetividade na implementação de algumas políticas antitabaco existentes no Brasil voltadas a reduzir a iniciação e/ou estimular a cessação dessa prática, como: (i) a presença de cigarros com aromas e sabores no mercado brasileiro, muito atrativos particularmente para adolescentes e jovens adultos. Tal situação acontece apesar de existir uma resolução da Anvisa de 2012 que a impediria ^9^, mas que sofreu interferência da indústria do tabaco a partir de uma ação de inconstitucionalidade ^28^, contribuindo para que, entre 2012 e 2021, triplicasse o número de registros de produtos derivados do tabaco contendo aromas e sabores junto à Anvisa ^29^; (ii) o preço muito barato do cigarro fabricado legalmente no Brasil, o segundo mais barato da região das Américas ^30^, e em queda no seu preço real desde 2017 ^15^^,^^17^^,^^30^; (iii) a forte presença de cigarros de origem ilegal no mercado brasileiro - 40% do total de cigarros consumidos em 2019, sendo essa uma das maiores proporções do mundo ^13^^,^^17^^,^^18^^,^^23^^,^^31^ -, vendidos a um preço inferior ao encontrado para os cigarros legalmente fabricados no país ^18^; (iv) a exposição abusiva/ilegal dos produtos derivados do tabaco, fora e dentro dos pontos de venda, muitas vezes por meio de painéis luminosos e coloridos e perto de balas e doces para atrair principalmente os menores de idade ^9^^,^^32^^,^^33^; e (v) a venda de produtos derivados do tabaco pela internet, o que é proibido ^9^^,^^34^.

Ademais, nos últimos anos, tem-se observado no país o aumento do consumo de outros produtos derivados do tabaco pelos jovens brasileiros, como o cigarro de palha e o narguilé ^32^^,^^35^^,^^36^. Isso se aplica também para os produtos que têm sua comercialização proibida no Brasil, apesar da forte pressão da indústria do tabaco para reverter tal situação ^37^, tais como os dispositivos eletrônicos para fumar, que são particularmente atraentes para a geração dos nascidos no século XXI ^36^. A literatura mostra que o uso de todos esses outros produtos derivados do tabaco favorece a iniciação ao consumo do cigarro tradicional e, consequentemente, a manutenção da dependência da nicotina ^33^^,^^36^^,^^38^.

Diante do cenário atual da política antitabagismo, provavelmente não por acaso, cerca de um em cada cinco jovens adultos fumantes de cigarro industrializado relatou ter adquirido cigarro avulso na sua última compra em 2019, o que representou 360 mil indivíduos entre 18 e 24 anos no Brasil. Tal achado é extremamente preocupante e contribui para que a indústria do tabaco seja bem-sucedida na sua estratégia de marketing voltada a substituir uma parte dos seus consumidores atuais, os quais irão inevitavelmente falecer ^39^, por uma nova geração que irá garantir que os lucros oriundos da venda dos produtos derivados do tabaco continuem ^30^^,^^32^^,^^38^^,^^39^. Atualmente, no Brasil, estima-se que 162 mil pessoas morram anualmente em consequência do uso atual (ou passado) dos produtos derivados do tabaco. A epidemia do tabagismo no Brasil envolve um custo de cerca de R$ 125 bilhões anuais, entre custos diretos para o sistema de saúde e indiretos para a sociedade, o que cobre apenas 10% do que a indústria do tabaco arrecada com impostos ^40^. É fundamental, portanto, que o Brasil implemente de forma efetiva as medidas relacionadas à redução de demanda e oferta de produtos derivados do tabaco ^1^ para avançar na diminuição da quantidade de dependentes de nicotina no país. Entre adolescentes e jovens adultos, as últimas pesquisas nacionais mostraram, por exemplo, uma proporção de fumantes estagnada no tempo e/ou com indicação de aumento ^10^^,^^11^^,^^12^^,^^13^^,^^36^. Em particular, o cenário encontrado neste estudo aponta para a necessidade de estimular os poderes federais, estaduais e municipais a promover ações educativas e de fiscalização, inclusive por meio de ações conjuntas com os órgãos do comércio varejista.

Existem poucos estudos internacionais que tenham estimado a proporção de compra de cigarros avulsos na população adulta de outros países ^6^^,^^21^^,^^41^^,^^42^. Enquanto a proporção de consumo de cigarros avulsos nos Estados Unidos parece ser menor do que a observada no Brasil (5,4% em 2014), outros países, como México (17% em seis cidades em 2008) e Índia (94% em quatro cidades em 2019), apresentam patamares mais elevados. Já a informação sobre a proporção de compra de cigarros avulsos nos últimos 30 dias entre adolescentes é bem mais abundante ^43^. Quando comparamos a proporção de compra de cigarros avulsos no último mês entre adolescentes brasileiros em 2019 (70%) ^19^ com os dados da pesquisa *Global Youth Tobacco Survey*, que compõe o sistema de monitoramento da epidemia do tabagismo proposto pela OMS nos países, coletados entre 2010 e 2018, percebemos que a proporção foi bem superior à observada entre o restante dos países da região das Américas (40,7%) ou à média mundial (37,1%) ^4^. As comparações entre as proporções encontradas para o Brasil, de 2008 a 2019, e aquelas identificadas para outros países devem ser feitas, contudo, com cautela, haja vista que esses índices são, obviamente, derivados não somente das características sociodemográficas e de comportamento de fumar da população em estudo, mas também do nível de implementação do conjunto das políticas antitabagismo e dos determinantes macrossociais corresponsáveis por um ambiente favorável à aceitação de ilegalidade ^1^^,^^26^.

Embora as análises do artigo sejam baseadas em pesquisas de representatividade nacional, os resultados podem estar sujeitos a viés de informação: (i) a informação autorreferida sobre a quantidade de cigarros adquirida na última compra pode ter sido subestimada devido à redução da aceitação do comportamento de fumar na sociedade ^43^^,^^44^; (ii) o fato de se avaliar a compra avulsa apenas entre indivíduos que relataram consumir cigarros industrializados não exclui a possibilidade de que uma parte dos informantes que fazem uso concomitante de outros produtos derivados do tabaco, tais como cigarro eletrônico ou cigarro de palha, tenha relatado a aquisição de cigarros avulsos desses outros produtos na última compra. Ambas as situações poderiam levar, portanto, a superestimar a proporção de consumo de cigarros industrializados avulsos para o período mais recente. Finalmente, as estimativas do consumo total de cigarros avulsos foram ponderadas pela quantidade de cigarros fumados em média por dia. O pressuposto de que a compra direta refletiria o modo padrão de obtenção de cigarro desse fumante pode fazer mais sentido para o período mais recente de aumento de acessibilidade econômica ao cigarro e/ou contexto favorável de aceitação da ilegalidade ^3^^,^^14^^,^^17^^,^^26^.

## Conclusão

A análise inédita da proporção de compra de cigarro avulso entre adultos no Brasil entre 2008 e 2019 reflete a importância de se buscar uma sinergia na implementação efetiva das medidas voltadas para a redução da iniciação e para o estímulo à cessação do fumo no país. Há motivo de preocupação quando percebemos que o fortalecimento da política tributária entre 2008 e 2013 foi acompanhado de um aumento na proporção de compra de cigarros avulsos. A preocupação continua quando constatamos que, apesar da queda do preço real do maço de cigarros a partir de 2017, um contexto favorável de impunidade e ilegalidade, aliado à baixa efetividade de medidas voltadas para redução da demanda e oferta dos produtos derivados do tabaco, levou provavelmente a um aumento na diferença da proporção de compra de cigarros avulsos entre jovens e adultos. A presença permanente do cigarro avulso como modalidade de aquisição possível contribui para que subgrupos populacionais mais vulneráveis do ponto de vista econômico se tornem e/ou permaneçam dependentes do comportamento de fumar e, consequentemente, sofram/venham a sofrer com os problemas de saúde a ele associados.
